# The Streets of Venice

**Published:** 1883-07

**Authors:** 


### THE STREETS OF VENICE.

Many persons are under a great misapprehension as to the means of transit or locomotion in Venice. It is a mistake to suppose that there are no streets, and that it is absolutely necessary to go from place to place by gondola. It is true that three bridges—the Rialto bridge of the Middle Ages, and two modern iron bridges—span the Grand Canal, which divides the city in equal halves ; it is true that the city is built upon one hundred and seventeen islands, intersected by one hundred and fifty small canals and two thousand four hundred and eighty passages; but almost every one of the water streets have a quay or foot-path bordering it, while four hundred bridges unite island to island, so that it is quite possible to go to every part of the city on foot, although few perhaps would care to do so, for there is not in all the world a more difficult place for the traveler, guided only by the "light of nature," to find a given spot. That spot may be only a few hundred yards away, but to reach it he may have to cross half a dozen bridges, some leading to the right, and some to the left, and traverse as many squares, of which there are three hundred and ninety-six, one hundred and twenty-seven larger squares, and two hundred and sixty-nine smaller squares.

Dr. Albert Day of Boston says that one of the chief predisposing causes of inebriety is civilization, entailing as it does in its modern development so great expenditure of nerve force. Its action in this way is seen more among those who live by brain work or are engaged in indoor occupations. He also holds that atmospheric influence is another undoubted exciting cause of this affection, paroxysm, for example, being caused by exposure to sea air, easterly winds, and other similar changes. It has been said that there is an inborn element in the nature of man which develops inebriety. "Could we trace the cause of this," he adds, "we should doubtless find that the whole race is tainted with this disease, coming down to us through the ages in obedience to the laws of heredity. Our fathers from earliest history were addicted to drunkenness."

